# Breast Cancer Diagnosis Using an Efficient CAD System Based on Multiple Classifiers

**DOI:** 10.3390/diagnostics9040165

**Published:** 2019-10-26

**Authors:** Dina A. Ragab, Maha Sharkas, Omneya Attallah

**Affiliations:** 1 Electronics and Communications Engineering Department, Arab Academy for Science, Technology, and Maritime Transport (AASTMT), Alexandria 1029, Egypt; msharkas@aast.edu (M.S.); o.attallah@aast.edu (O.A.); 2 Electronic and Electrical Engineering Department, University of Strathclyde, Glasgow G1 1XW, UK

**Keywords:** the computer-aided detection, the pectoral muscle removal, the statistical features, the decision trees, the k-nearest neighbor, feature selection

## Abstract

Breast cancer is one of the major health issues across the world. In this study, a new computer-aided detection (CAD) system is introduced. First, the mammogram images were enhanced to increase the contrast. Second, the pectoral muscle was eliminated and the breast was suppressed from the mammogram. Afterward, some statistical features were extracted. Next, k-nearest neighbor (k-NN) and decision trees classifiers were used to classify the normal and abnormal lesions. Moreover, multiple classifier systems (MCS) was constructed as it usually improves the classification results. The MCS has two structures, cascaded and parallel structures. Finally, two wrapper feature selection (FS) approaches were applied to identify those features, which influence classification accuracy. The two data sets (1) the mammographic image analysis society digital mammogram database (MIAS) and (2) the digital mammography dream challenge were combined together to test the CAD system proposed. The highest accuracy achieved with the proposed CAD system before FS was 99.7% using the Adaboosting of the J48 decision tree classifiers. The highest accuracy after FS was 100%, which was achieved with k-NN classifier. Moreover, the area under the curve (AUC) of the receiver operating characteristic (ROC) curve was equal to 1.0. The results showed that the proposed CAD system was able to accurately classify normal and abnormal lesions in mammogram samples.

## 1. Introduction

Nowadays, breast cancer is one of the most common cancers in women. According to the World Health Organization (WHO), the number of cancer cases expected in 2025 will be 19.3 million. Although the rates of women with breast cancer are increasing tremendously, recently death rates from breast cancer have reduced. This is due to the advances made in medical imaging, image processing and machine learning techniques, which have enabled radiologists to identify cancer in the early stages. Therefore, the early detection of breast cancer is essential. The early detection of breast cancer can improve the quality of diagnosis and follow up planning. It also reduces the death rates among women, as according to statistics, 96% of cancers are curable in the initial stages. 

Mammography is a widely accepted method to diagnose breast cancer at its early stage [1]. Mammogram images have different views when scanned from different angles. Among these views are the mediolateral-oblique view (MLO) and the craniocaudal (CC) view. The MLO view of the breast projects more breast tissue than that of the CC view. This is because of the slope of the chest wall. Several signs are commonly used to detect breast cancer from mammograms. Among them are the masses, microcalcifications (MCs), and architectural distortions. The former two symptoms are very important indicators of the tumor in the primary stage. The architectural distortion indicators of breast cancer have been found to be less significant than masses and MCs [2].

Computer-aided detection systems (CAD) using machine learning techniques can be used for mammogram images for the early detection of breast abnormalities, diagnosis and classification of tumors. The CAD may assist radiologists in medical diagnoses with great accuracy and reliability. Moreover, CAD may prevent human-based diagnostic errors that are caused by visual fatigue and the effort made during an examination. 

A CAD system consists of several modules which are image enhancement, image segmentation, feature extraction, feature selection (FS), and feature classification. 

Recently, several researchers studied and proposed methods for breast abnormality classification in mammography images. 

Image segmentation is an important step in the CAD system. The presence of the pectoral muscle, which is located at the upper right or left side of the breast, may disturb the detection of breast cancer. This is because it appears with a similar density as the dense tissues in the mammogram image. Therefore, several authors have tried to eliminate pectoral muscle and segment the breast from the mammogram with several segmentation techniques. Alam et al. [3] used the k-means clustering to eliminate the triangular area of the pectoral muscle. Abdellatif et al. [4] used the normalized graph cuts to delineate the pectoral muscle. Nagi et al. [5] used the morphological preprocessing and the seeded region growing (SRG) algorithm to remove the noise, suppress the artifacts and remove the pectoral muscle. Shah [6] used a triangular mask to the upper left corner of the mammogram for pectoral muscle detection and suppression.

For the feature extraction and classification steps, the following authors used some popular techniques. Sharkas et al. [7] used the discrete wavelet transform (DWT), the contourlet transform and the principal component analysis (PCA) methods for the feature extraction. The system was able to detect and classify normal and abnormal tissues in addition to benign and malignant MC tumors of the digital database for screening mammography (DDSM) [8]. The achieved rate was almost 98 %. Ragab et al. [9] used the DWT as a feature extraction technique to detect mass abnormalities in the breast. In addition, a comparison between support vector machines (SVM) and artificial neural networks (ANN) for classifying normal, abnormal tissues, benign, and malignant MCs tumors was introduced. The authors achieved a classification accuracy of 96% and 98% for ANN and SVM, respectively. Gunawan [10] presented a new method, which depends on wavelet transform to calculate statistical features; the classification accuracy was 96%. Al Sharkawy, M. et al. [11] detected mass lesions using the DWT and SVM and the rate achieved was 92%. The authors in [12] extracted some statistical features such as the mean, variance, range, standard deviation, and entropy from the mammogram. They performed their experiments on 15 samples (5 normal, 5 benign, and 5 malignant cases) extracted from the mammographic image analysis society digital mammogram database (MIAS) [13]. They classified the samples according to the pixel intensity statistical feature [12]. Fu et al. [14] extracted 61 features from both the spatial domain and spectral domain. Beura et al. [15] used the gray level co-occurrence matrix (GLCM) and DWT to extract the texture features from the region of interest (ROI) image. Pawar and Talbar [16] used a wrapper method for feature selection. The features were extracted using the wavelet co-occurrence features from the four decomposition levels. Mohanty et al. [17] used the contourlet transform to extract the features from cropped mammogram ROI images.

Furthermore, feature selection (FS) is an important process in machine learning. It is widely used in medical applications to reduce the dimensionality of the dataset and remove redundant and irrelevant features. Further, FS selects only the features, which can influence the classification results of a predictive model. Several FS techniques have been studied and proposed for breast cancer classification tasks. Fu et al. [14] used a sequential forward search (SFS) algorithm which was used followed by SVM and general regression neural network (GRNN) classifiers. After selecting the most relevant features, the reported technique achieved an area under the curve (AUC) of 0.9800 and 0.9780 for SVM and GRNN, respectively. Beura et al. [15] performed feature selection using two simple filter methods namely, the *t*-test and F-test to get the relevant features. Moreover, the back propagation neural network (BPNN) was used as a classifier. The accuracy obtained for the two standard datasets MIAS [13] and DDSM [8] were 98.0% and 98.8%, respectively. Pawar and Talbar [16] proposed a genetic fuzzy FS approach which results in an accuracy of 89.47%. Mohanty et al. [17] proposed an FS algorithm named forest optimization. The different classifiers such as SVM, k-nearest neighbor (k-NN), Naïve Bayes, and C4.5 were used to classify normal and abnormal mammogram lesions. The highest accuracy achieved was 99.08 % for the C4.5 classifier using the DDSM dataset. The authors used the MIAS dataset as well and achieved an accuracy of 97.86% for the Naïve Bayes classifier [17]. 

From the literature study, it was noticed that the pectoral muscle removal, feature extraction, selection, and classification are the key modules to increase the accuracy of a CAD system. The literature focused on using several datasets separately to perform a CAD system. To the authors’ own knowledge, no one has tried combining more than one dataset to construct a CAD system. In addition, most research has focused on using individual classifiers to construct a CAD system. In addition, the effect of using multiple classifier systems with different structures combined with feature selection on classification accuracy was not extensively studied. 

In this paper, a new CAD system to classify normal and abnormal mass lesions from mammogram images is presented. The paper focuses on combining two common datasets to construct a CAD system. It uses individual classifiers for the classification stage. In addition, it uses ensemble classifiers and compares the performances of these ensemble classifiers with the performance of individual classifiers. In addition, the paper performs an augmentation process to increase the size of the datasets and attempts to improve classification results. The proposed CAD system consists of four steps: image enhancement, segmentation, feature extraction, and classification. A suggested modification to the CAD system is introduced by adding feature selection before classification to improve accuracy. In the image enhancement step, the images were enhanced using the adaptive contrast enhancement technique. In the segmentation step, the pectoral muscle was eliminated and the breast was suppressed from any artifacts. Afterward, in the feature extraction step, some statistical features were extracted. Next, the classification step was carried out using individual classifiers such as the k-NN, J48 decision tree (DT), random forest (RF) DT, and random tree (RT) DT. Additionally, several multiple classifier systems (MCSs) were constructed. It is noted that MCS usually outperform individual classifiers. For this reason, several types of MCS such as Adaboosted and bagged k-NN, J48 DT, RF DT, and RT DT classifiers were developed and their performances were compared with their individual classifiers. Moreover, an MCS was built using a combination of J48 DT, RF DT, and RT DT classifiers fused together using averaging fusion method. By attempting to increase the classification accuracy, two wrapper FS approaches based on best first and random search strategies were applied to the extracted features in order to select those features that improve the classification accuracy. 

The paper is organized as follows: Section 2 describes the CAD system, Section 3 shows the experimental setup and Section 4 shows the computed results of the technique. A discussion of the suggested technique is presented in Section 5, and finally, the work is concluded in Section 6.

## 2. Methodology

**Figure 1:**
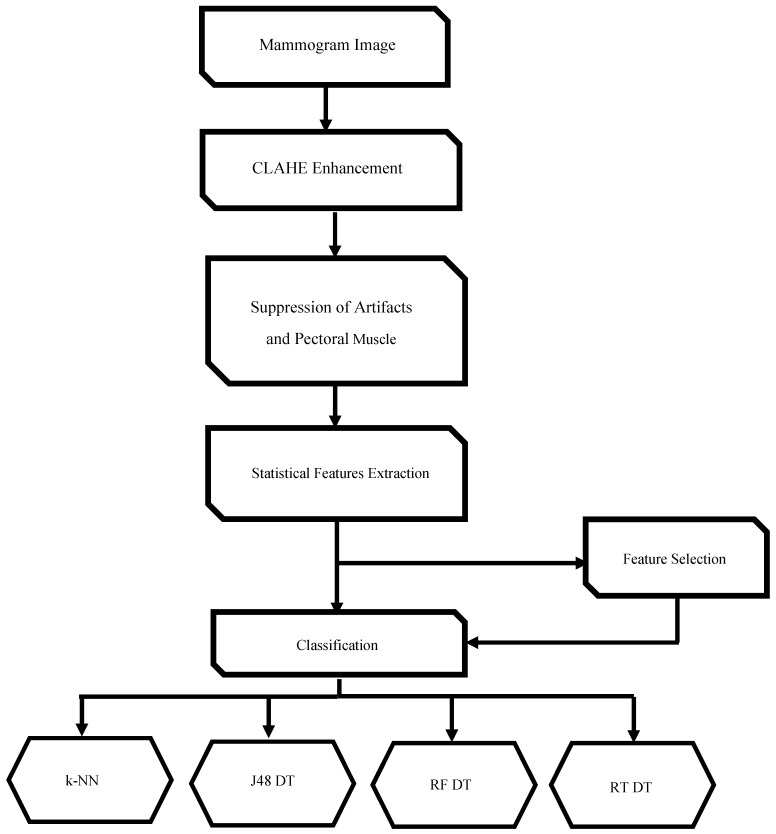
The block diagram of the computer-aided detection (CAD) system used.

As stated previously, a CAD system consists of several modules, which are (1) image enhancement, (2) image segmentation, (3) feature extraction, (4) feature selection (FS), (5) classification, and (6) an evaluation of the classifiers [18,19]. This proposed CAD system enhances images using a contrast enhancement method named contrast-limited adaptive histogram equalization (CLAHE) [20]. Afterward, it removes the pectoral muscle and suppresses the breast from any artifacts in the mammogram. Next, some statistical features are extracted and used to construct several individual and ensemble classification models. Finally, two wrapper feature selection techniques were used to select those features that improve the classification accuracy of both the individual classifiers and ensemble classifiers. The flow chart of the proposed CAD system used in this work is illustrated in Figure 1. 

### 2.1. Image Enhancement

**Figure 2:**
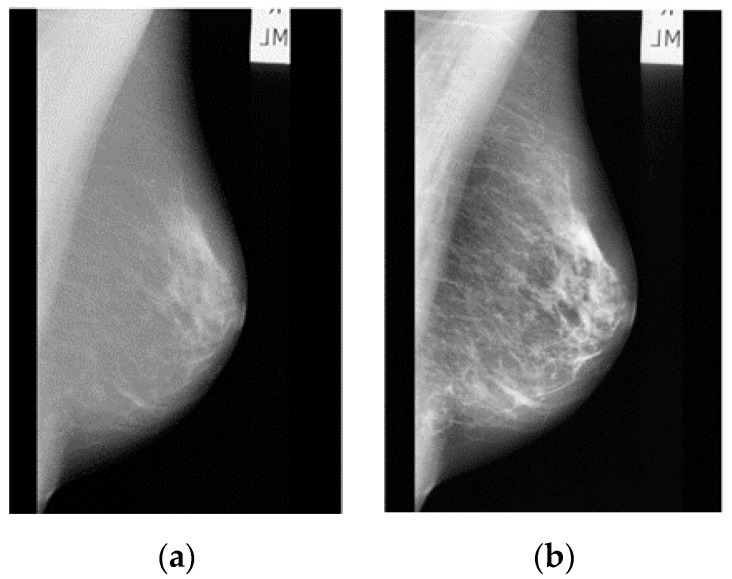
(**a**) Original abnormal mass case extracted from the Mammographic Image Analysis Society (MIAS) dataset [13] and (**b**) Enhanced image using contrast-limited adaptive histogram equalization method (CLAHE).

Image enhancement in this context means the processing of the images to increase their contrast and suppress noise in order to aid radiologists in detecting the abnormalities. There are many image enhancement techniques and among them is the adaptive contrast enhancement method (AHE). The AHE technique is capable of improving local contrast and bringing out more details in the image. It is an excellent contrast enhancement method for both natural and medical images [21,22]. In this paper, the contrast-limited adaptive histogram equalization method (CLAHE) which is a type of AHE was used to improve the contrast of images [21,22]. An enhanced image using CLAHE is shown in Figure 2. 

The CLAHE algorithm can be summarized as follows; [20]

1. Divide the original image into contextual regions.
2. Obtain a local histogram for each pixel.
3. Limit this histogram based on the clip level.
4. Redistribute the histogram using binary search.
5. Obtain the enhanced pixel value by histogram integration.

### 2.2. Image Segmentation

Image segmentation is used to divide an image into parts having similar features and properties. The main aim of segmentation is simplification, to represent the image in an easy analyzable way. In this paper, the region of interest (ROI) was extracted from the original mammogram image by suppressing the whole breast excluding the pectoral muscle and any other artifacts. Pectoral muscles are the triangle shape region located in one side of the MLO view of the mammogram either at the left or at the right top corner. The pectoral muscles appear approximately with a similar density as the dense tissues in the mammogram image. Therefore, removing the pectoral muscle plays an important role in detecting the tumor cell precisely. 

**Figure 3:**
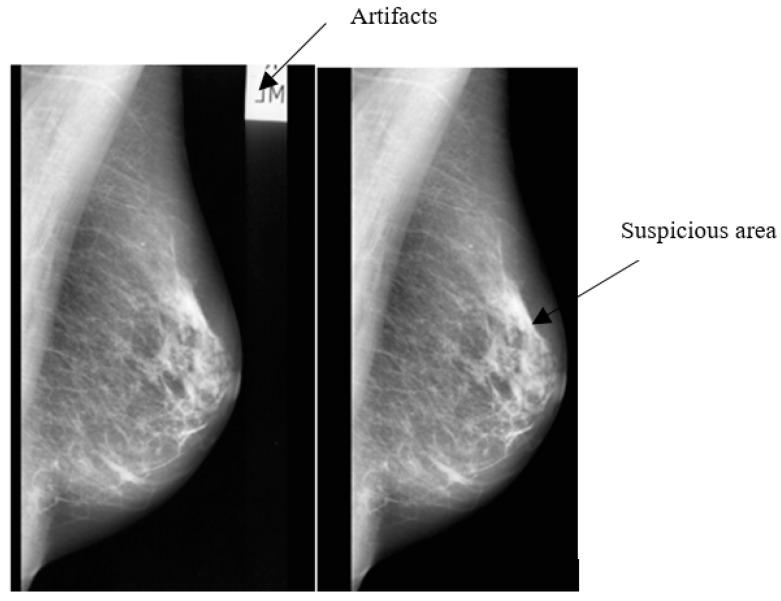
(**a**) Original enhanced abnormal mass case extracted from MIAS dataset [13] and (**b**) Suppressed image from artifacts.

The steps for the ROI segmentation can be summarized as follows:

1. Orient all the mammogram samples in the same direction. This step is done to avoid the situation of applying different methods for the left and the right-oriented MLO mammograms. Therefore, flip all the RMLO view to look like the LMLO view samples as an example.
2. Eliminate the mammogram image from any radiopaque artifacts, such as labels. This is performed by using thresholding and morphological operations [5]. A global threshold with a value of 18 was found to be the most suitable threshold for transforming the grayscale images into binary (0,1) format [5]. Figure 3 shows the mammogram image with artifacts suppression.
3. Remove the pectoral muscle using the seeded region growing (SRG) technique [5,23]. The SRG performs image segmentation with respect to a set of points, known as seeds [24].

**Figure 4:**
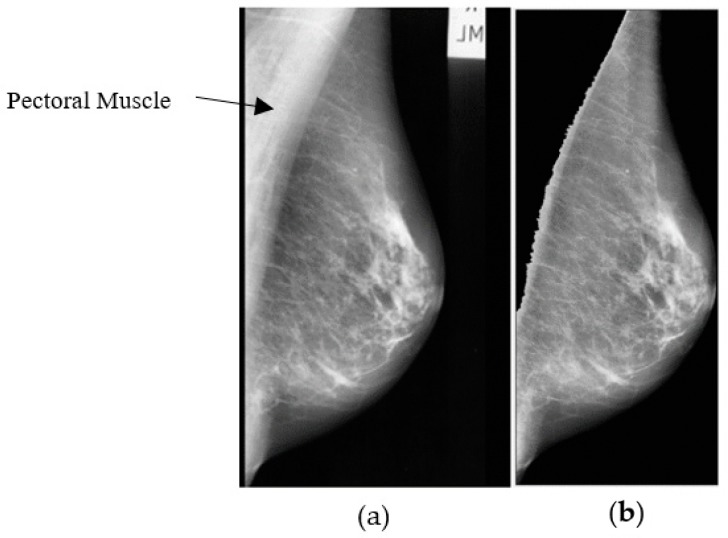
(**a**) Original enhanced abnormal mass case extracted from MIAS dataset [13] and (**b**) Pectoral muscle removal.

Figure 4 shows the mammogram image after removing (blacking) the pectoral muscle.

### 2.3. Feature Extraction

In this step, each image was divided into blocks of size 16 × 16. Afterward, some statistical features were calculated from each block of an image in the spatial domain. Next, the mean of these statistical features was calculated for each image in the dataset. Finally, all the features were combined in one feature vector. These features include entropy, mean, variance, standard deviation, range, minimum, maximum, and root mean square (RMS). 

#### 2.3.1. Entropy

Entropy is a statistical measure of randomness that can be used to characterize the texture of the input image. It can be used to describe the distribution variation in a region as well [12]. For an image I of size M × N, each image is split into Z blocks. Each Z block is of size F × G. The entropy for each block is calculated using Equation (1),

(1) $$Ent_{z}=-\overset{n-1}{\underset{i=0}{\sum }}pr_{i}\times \log pr_{i}$$

where *n* is the number of grey levels. $pr_{i}$ is the probability of a pixel having gray level *i*. 

The mean of the entropy for the *Z* blocks is calculated using Equation (2),

(2) $$Entropy=\frac{1}{Z}\overset{Z}{\underset{z=1}{\sum }}Ent_{z}$$

where *Z* is the total number of blocks in an image and *z* is the block order. 

#### 2.3.2. Mean

The mean for each image block *z* is calculated using Equation (3),

(3) $${µ}_{z}=\frac{1}{FG}\overset{FG}{\underset{i,j=1}{\sum }}p_{z}\left(i,j\right)$$

where *p_z_(i,j)* is the pixels value in the image block *z*, F × G is the size of each block *z*.

The mean of the mean for all Z blocks is calculated using Equation (4),

(4) $$Mean=\frac{1}{z}\overset{Z}{\underset{z=1}{\sum }}{µ}_{z}$$

#### 2.3.3. Variance

The variance (σ^2^) is the estimate of the mean square deviation of the grey pixel value from its mean value. It describes the dispersion within a local region [12]. The variance of each image clock *z* is calculated using Equation (5),

(5) $$\sigma_{z}^{2}=\frac{1}{FG}\overset{FG}{\underset{i,j=1}{\sum }}{\left(p_{z}\left(i,j\right)-\mu_{z}\right)}^{2}$$

The mean of the variance is determined using Equation (6),

(6) $$Variance=\frac{1}{Z}\overset{Z}{\underset{z=1}{\sum }}\sigma_{z}^{2}$$

#### 2.3.4. Standard Deviation

The standard deviation (σ) is the square root of the variance. The standard deviation for each block *z* of an image is calculated using Equation (7),

(7) $$\sigma_{z}=\sqrt{\frac{1}{FG}\overset{FG}{\underset{i,j=1}{\sum }}{\left(p_{z}\left(i,j\right)-\mu_{z}\right)}^{2}}$$

The mean of the standard deviation (sd) for all blocks Z of an image is calculated using Equation (8),

(8) $$Sd=\frac{1}{Z}\overset{Z}{\underset{z=1}{\sum }}\sigma_{z}$$

#### 2.3.5. Range

The range is defined by the formula given in Equation (9),

(9) $$R_{z}=p_{z}{}_{max}-p_{z}{}_{min}$$

where $p_{z}{}_{max}$ and $p_{z}{}_{min}$ are the maximal and minimal pixel values in an image block *z*. 

Then, the mean of the range is calculated using Equation (10),

(10) $$Range=\frac{1}{Z}\overset{Z}{\underset{z=1}{\sum }}R_{z}$$

#### 2.3.6. Root Mean Square

The root mean square (RMS) provides the arithmetic mean of the squares of the mean values along each row and column in a block z in an image. The mean of the RMS given by (µ RMS) of all blocks in an image of size F × G is given by (11).

(11) $$µRMS=\frac{\sqrt{\sum_{i=1}^{F}{\left|{µ}_{i,j}\right|}^{2}}}{F}$$

### 2.4. Classification

In this step, several classification models are constructed to classify the ROI to either normal or abnormal masses. This step is done using individual classifiers or multiple classifiers system (MCS). 

#### 2.4.1. Individual Classifiers

An individual classifier means that the classification process is done using only one classifier. For this purpose, k-nearest neighbor (k-NN) and several types of decision tree classifiers such as (J48), random forest (RF), and random tree (RT) are constructed [25,26,27,28].

1. K-NN is a popularly used classifier due to its simplicity, straightforwardness and high efficiency even with noisy data [29]. Even with its straightforwardness, it is capable of achieving high accuracy rates in medical applications [30,31]. K-NN assigns a class to each data point in the test set according to the class of it amongst k-nearest neighbors inside the training set [32]. This is done by measuring the distance between each data point in the test set needed to be classified and other data points in the training set. The distance indicates how similar the instance is in the test set to instances in the training set. The distance used in our approach is the Euclidean distance and the value of k used is two.
2. Decision Trees (DT) classifiers are commonly used in machine learning techniques. They are used extensively in medical applications such as breast cancer, ovarian cancer and heart sound diagnosis [33,34]. This is due to their ability to visualize reactions between data attributes. Visualization facilitates the doctors’ understanding of how the classification decision is made and an association between features in the data. A DT can handle categorical and numeric attributes. These classifiers are also robust to outliers and missing values. A DT classifies data points in the training set based on rules or conditions to form a tree structure. A DT construction is like a tree with a root node whose leaves represent class labels and branch nodes, which represent attribute and reasons, which lead to those class labels. Nodes are connected by arcs, which represent the conditions on the attributes. The attribute splitting is determined by a metric such as information gain, gain ratio, or Gini index. The DT has several types of trees such as J48, random forest (RF), and random tree (RT).

The J48 classifier uses a top-down and greedy search through all probable nodes to construct a DT. The RF is considered a strong classifier that achieves high classification accuracy with datasets with a large number of features even without any feature selection. Moreover, RF is capable of determining the important attributes of a dataset. Several trees are built to select the best tree on the split. The random tree is a DT classifier, which chooses a random number of attributes to construct a DT and classify the data [33,34].

#### 2.4.2. Multiple Classifier System (MCS)

The multiple classifiers system (MCS) is a hybrid method that fuses classification results of a number of classifiers connected together by a combiner. The MCS adds the strength of each classifier that usually exceeds the performance of each individual classifier. The MCS can avoid the possibility of poor results that are generated from a certain unsuitably selected model. This is equivalent to medical applications in cases where diagnosis to a specific illness is made by taking decisions from various doctors to come to a more confident final decision [35]. 

The MCS has two structures: parallel and cascaded. In the parallel structure, a number of classifiers are connected in parallel and their predictions are fused using either, majority voting, maximum probability, minimum probability, or averaging methods. These classifiers may be of different types or the same type, such as the bagging ensemble. The bagging ensemble stands for bootstrap aggregation. It depends on the bootstrap resampling method to generate a number of data subsets from the original data randomly. These subsets are used to build several classifiers of the same type, such as decision trees or k-NN’s. In the cascaded structure, the classifiers are connected in a cascaded manner. This structure includes the Adaboosting. The Adaboosting ensemble consists of a number of classifiers connected in series. Each classifier in the ensemble attempts to improve the performance of the previous weaker classifier. It uses a class weighting resampling technique to train the next classifier in the ensemble. Instances that are not correctly classified with the first classifier in the ensemble are given higher weights and then these resampled instances enter the next classifier. This procedure is repeated until all classifiers of the ensemble are processed.

In this paper, different MCS of different structures were constructed to classify the ROI to normal or abnormal. The first structure includes bagging with k-NN, J48 DT, RF DT, and RT DT. The second structure is Adaboosting with k-NN, J48 DT, RF DT, and RT DT. The third structure is an MCS constructed with k-NN, J48 DT, RF DT, RT DT and combined using averaging fusing technique.

### 2.5. Feature Selection

Feature selection (FS) is commonly used in medical image processing, as it reduces the time needed and the effort made by physicians to measure irrelevant and redundant features. It could avoid overfitting that might occur during the learning process of the predictive model. It may also lower its complexity and speed up the prediction process [36]. It is divided into three approaches: filter, wrapper, and embedded. The former is the simplest and fastest method. It uses criteria or metric for choosing variables, which is independent of the classification process. The wrapper is a classifier dependent FS method and is more complex and slower than a filter. However, it involves the predictive model in choosing variables, which are usually preferred. In the embedded method, the FS process is inserted within the classifier structure. The embedded method includes the interaction with the classification model, while at the same time being far less computationally intensive than the wrapper methods [36].

Further, FS uses a search strategy to generate a subset of features that are then evaluated using a metric. There are several searching strategies for FS, however, in this paper, two strategies are applied to generate two wrappers FS approaches: (1) Best First (BF) and (2) Random search. The BF searches the space of attribute subsets by a greedy heuristic method. The BF can begin searching with an empty set of attributes and search forward, backward, or in both directions. It has backtracking so if a track that is investigated looked less favorable, the BF method can backtrack to a more favorable previous subset and continue the search from there. The random search examines feature space in a random manner. It can begin with a random feature or specified feature and add features randomly to get the best subset found [37,38,39]. 

In this paper, the random search with random initial point and BF search with bi-direction tracking were applied to select and reduce the complexity of the classification models and select the features, which improve the classification accuracy.

### 2.6. Evaluation

There are several evaluation metrics used in this paper to evaluate a classifier performance. Among them is the accuracy, the receiver operating curve (ROC), the area under ROC curve (AUC), sensitivity, specificity, precision, and the F1 score. 

#### 2.6.1. Accuracy

Accuracy is the measure used to determine how many instances the classifier has correctly classified from the whole data. Thus, it indicates the ability of the classifier to perform well. The accuracy is defined as in Equation (12).

(12) $$accuracy=\frac{TP+TN}{TN+FP+FN+TP}$$

where, TP is the true positive, which is the number of positive class instances that are correctly classified and TN is the true negative, which is the number of negative class instances that are correctly classified. Whereas, FP is the false positive which is the number of negative class instances that are incorrectly classified as positive class and FN is the false negative, which is the number of positive class instances that are incorrectly classified as negative class.

#### 2.6.2. The Receiver-Operating Characteristic 

The receiver operating characteristic (ROC) analysis is a well-known evaluation method for detection tasks. It is based on a statistical decision theory and it is developed in signal detection theory. The ROC analysis was first used in medical decision making and subsequently, it was used in medical imaging. A ROC curve is a graph representing the true positive rate (TPR) as a function of the false positive rate (FPR). The TPR is called sensitivity or recall while the true negative rate (TNR) is called the specificity and they are defined as in Equations (13) and (14) [40].

(13) $$sensitivity\;\left(Recall\right)=\frac{TP}{TP+FN}$$

(14) $$specificity\;\left(TNR\right)=\frac{TN}{TN+FP}$$

#### 2.6.3. The Area under the ROC Curve 

The area under the ROC curve (AUC) is used in medical diagnosis systems. The AUC provides an approach for evaluating models based on an average of each point on the ROC curve. Thus, the AUC score is always between 0 and 1 for a classifier performance and the model with a higher AUC value gives a better classifier performance [41]. 

#### 2.6.4. Precision

Precision is the ratio of correctly predicted positive observations of the total predicted positive observations. High precision relates to the low FPR. Precision is calculated using the following Equation [42,43],

(15) $$Precision=\frac{TP}{TP+FP}$$

#### 2.6.5. F1 Score

The F1 score is the harmonic mean of precision and recall. It is used as a statistical measure to rate the performance. In other words, an F1 score is from 0 to 9, where 0 being lowest and 9 being the highest. Therefore, this score takes both false positives and false negatives into account. The F1 score is defined as in Equation (16) [42,43].

(16) $$F1\;score=\frac{2*Recall*Precision}{Recall+Precision}$$

## 3. Experimental Setup

The proposed CAD system was applied to mammogram images providing a possibility of each image to belong to one of the two classes, either normal or abnormal. In this work, a computationally efficient tool called Waikato Environment for Knowledge Analysis (WEKA) [44] was used. WEKA is an open-source software, which consists of a collection of machine learning algorithms for data mining tasks.

### 3.1. Dataset Description

In this study, two datasets were used to test the performance of the proposed CAD system. These datasets are named (1) the mammographic image analysis society digital mammogram database (MIAS) [13] and (2) The Digital Mammography Dream Challenge [45]. The description of each dataset is discussed in this section. 

An organization of the UK research groups, called Mammographic Image Analysis Society (MIAS), created a database of digital mammograms. The films have been digitized to a 50-micron pixel edge. All images are available with a size of 1024 × 1024. Mammogram images are available via the Pilot European Image Processing Archive (PEIPA) at the University of Essex. The MIAS dataset has 322 annotated images of left and right breasts. The MIAS dataset [13] was chosen to verify the proposed CAD system.

Digital Mammography Dream Challenge [45] is a new dataset and is used to obtain more training samples. It is one of the larger efforts in using artificial intelligence to attempt to improve breast cancer screening outcomes. This crowdsourcing coding competition offers a large monetary prize for the best algorithm for predicting breast cancer on screening mammography. Final Challenge results were expected in late 2017 with open access to the winning coding algorithms. Hence, ongoing short to intermediate-term activities focus on improving this open-source algorithm to achieve higher accuracy, building on the challenge results to bring artificial intelligence products to the market and exploring how an accurate algorithm might be potentially incorporated into screening practice [46]. This dataset consists of 34 and 466 abnormal and normal samples, respectively. 

### 3.2. Data Augmentation

Generally, training on a large number of training samples performs well and gives high accuracy rates. However, the biomedical datasets contain a relatively small number of samples due to limited patient volume. Consequently, data augmentation is essential. Data augmentation is a method for increasing the size of the input data points by generating new data points from the original input data. There are many strategies for data augmentation. The one used in this study is the rotation and the flipping methods.

The total number of normal samples in the digital mammography dream challenge dataset was 466, but only 300 samples were selected. As for the abnormal, the total number of samples was only 34. Therefore, by comparing to the number of normal samples, there was no balance between the two classes. Therefore, firstly, the samples were rotated by 0, 90, 180 and 270 degrees. Then, each rotated image was right flipped. Therefore, each sample of the abnormal class was augmented to eight images. As a result, the number of abnormal samples used became 34 × 8, which is equal to 272 samples as demonstrated in Table 1. 

Moreover, when using the samples of the MIAS dataset, the number of normal samples were more than that of the abnormal ones: 120 and 93 samples were selected from the normal and abnormal classes, respectively. However, this time the samples of both classes were augmented once using the rotation method. The number of the normal class became 120 × 4 = 480 samples and the number of abnormal class became 93 × 4 = 372 samples as shown in Table 1. 

On the other hand, when combining the two datasets together, for the abnormal samples, all the samples of the two datasets were used. Therefore, the total samples became 127 samples. While for the normal samples, 68 samples were selected from the digital mammography dream challenge dataset and 132 samples from the MIAS dataset so the total for this class became 200 samples. Only one augmentation technique, which was the rotation, was applied for each class. Therefore, each image was augmented to four images giving 800 and 508 for normal and abnormal classes, respectively as illustrated in Table 1.

## 4. Results

The samples were enhanced and segmented using the method mentioned in Section 2. Some statistical features were extracted from the segmented samples after splitting each image into blocks. Furthermore, the mean of each extracted feature was calculated. Therefore, each feature became a one-dimension vector. Finally, all the features were combined together in one feature vector. Then, these features were used to construct a classification model using both individual and multiple classifiers. Moreover, the two wrapper FS methods were employed to select which features influence classification accuracy. The two wrapper FS methods were based on the best first and random search methods. The accuracy, AUC, sensitivity, specificity, precision, and F1 score were calculated for each classifier. 

All the results were verified using a fivefold cross-validation, which split the data using an 80-20% ratio. For k-NN, the number of k was chosen using a five-nested cross-validation, which resulted in the best k chosen being equal to two. A nested cross is a well-known procedure. It is used to overcome over-fitting and over-optimistic results that may occur during model construction, parameter and feature selections. The Euclidean distance was used as a distance metric for a k-NN classifier. For decision trees, nested cross-validation was also used to reduce error pruning. For the random forest, the number of trees generated was 10. For Adaboosting and the bagging classification, the number of ensemble classifiers was 10.

The proposed methods were applied to the MIAS and the digital mammography dream challenge datasets separately. Each sample of the MIAS dataset was augmented to four images, while the samples of the digital mammography dream challenge dataset were augmented to eight images as in Table 1.

The classification results of the individual and MCS using all classifiers, the best first and the random search FS for the MIAS and the digital mammography dream challenge datasets are illustrated in Table 2, Table 3, Table 4, Table 5, Table 6, and Table 7, respectively. 

Moreover, the MIAS and the digital mammography dream challenge datasets were combined together and all the samples went through the same procedures as previously stated. Table 8 shows a comparison between the classification results of the individual classifiers, their ensembles, and the MCS constructed using all these classifiers for the combined datasets. 

Additionally, the two wrapper FS methods were employed to select which features influence classification accuracy.

Table 9 and Table 10 show the results of the individual, MCSs obtained after the best first and the random search wrapper feature selection approaches. 

In comparison to other researches results, the results obtained from the newly proposed methods were the highest results. This is clear in Table 11.

## 5. Discussions

This paper presents a new CAD system to differentiate between normal and abnormal mass lesions in the breast. In the segmentation step, the breast was segmented from any artifacts and the pectoral muscle was eliminated. Afterward, each image was split into blocks of size 16 × 16. Some statistical features were extracted from these blocks such as the entropy, mean, standard deviation, minimum, maximum, variance, range, and RMS. Afterward, the mean was calculated for each feature. Finally, all the features were combined together to have a feature vector of only eight features. These features were then used to construct several individuals and MCS models such as the decision tree (J48), random forest (RF), random tree (RT), k-NN, and their ensembles. Additionally, some features were selected using two FS techniques: (1) best first and (2) random search to increase the classification accuracy of both individual and MCS models of different structures.

The nested cross fold validation was conducted for the optimization of the k for k-NN and prune over fitting of decision tree classifiers. Then, the cross-fold validation for actual validation was used. Moreover, the nested cross-fold validation was employed for feature selection. First, the cross-fold validation was used for selecting the features through nested folds, and then it was used to validate the results of the features selected and evaluate the classifier performance based on these features.

The experiments were applied on the MIAS and the digital mammography dream challenge datasets. First, each set was trained and tested separately, and then both datasets were merged to study the effect of combining two datasets on classification accuracy. 

To increase the number of sample data, augmentation was applied to the samples. In this work, all images of both datasets were rotated by 0, 90, 180 and 270 degrees. Moreover, the flipping method was applied to the abnormal samples of the digital mammography dream challenge dataset. This was necessary due to the very small size of the abnormal samples compared to the normal ones. Therefore, the samples were first rotated, and then each rotated sample was flipped horizontally. Hence, each image was augmented to eight images. 

**Figure 5:**
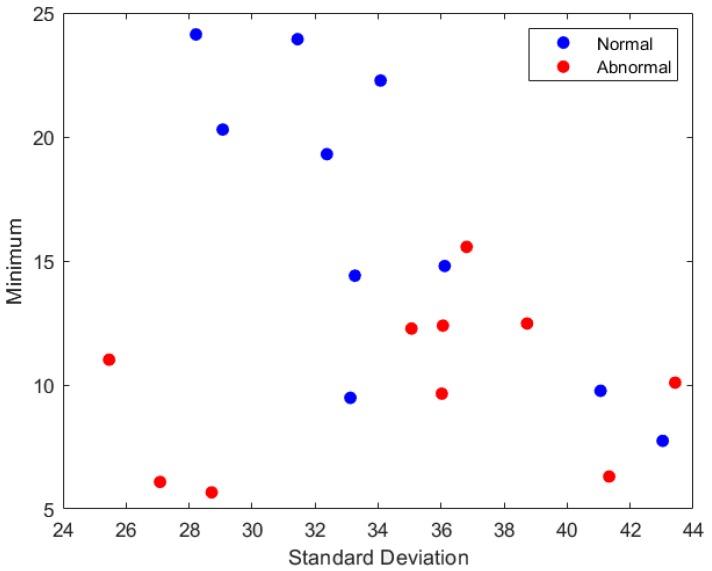
The minimum feature values versus the standard deviation feature values for the first 10 samples of images and their rotated versions.

**Figure 6:**
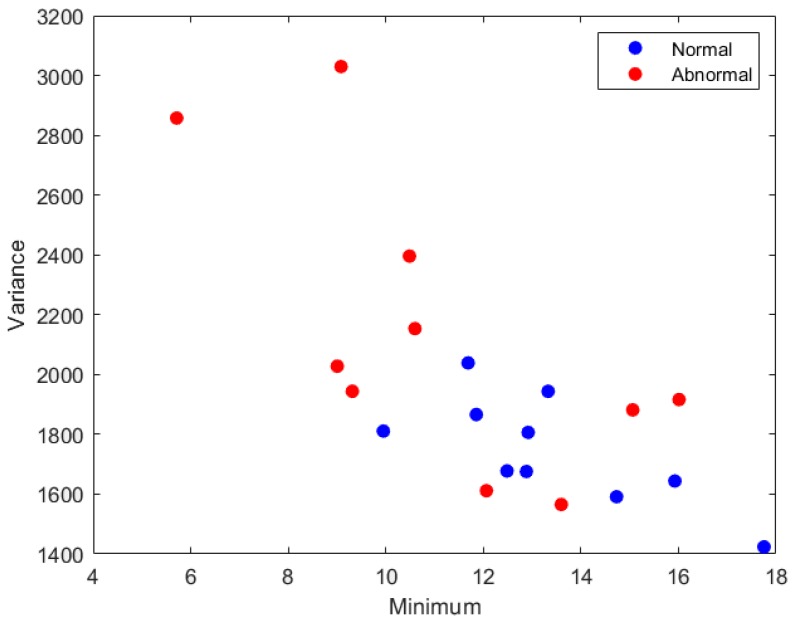
The variance feature values versus the minimum feature values for the second 10 samples of images and their rotated versions.

All the features calculated for the images and the rotated ones were the same, except for the range feature. Table 12 shows the name of the features that were omitted using each FS method. It is clear from the table that the range feature was usually omitted using most FS strategies. Figure 5 and Figure 6 show the two-dimensional scatter plots based on the feature vectors for normal and abnormal samples of breast cancer datasets. Figure 5 represents the standard deviation feature versus the minimum feature for the first 10 samples of images and their orientations with a total of 40 images for each class. Figure 6 represents the variance feature versus the minimum feature features for the second 10 samples and their orientations.

For the MIAS samples, the total number of samples used was 852 as shown in Table 1. When classifying normal and abnormal lesions using individual classifiers and their ensembles, it was clear that the Adaboosting of J48 DT and the random forest achieved the highest accuracy of 100%. This was the highest accuracy compared to the other classifiers as shown in Table 2. Moreover, the AUC, the sensitivity, specificity, precision, and the F1 score for both classifiers also achieved the highest scores. All of them recorded a value of one (100%). 

Moreover, when using multiple classifiers, the Adaboosting ensemble of k-NN, J48 DT, RT DT, and RF DT proved to have the highest accuracy, AUC, sensitivity, specificity, precision, and F1 score compared to the bagging ensemble as shown in Table 2. 

Furthermore, when selecting the features using the best first searching strategy as shown in Table 3, the numbers of features selected were 5, 6, 7, and 6 for k-NN, J48 DT, RF DT, and RT DT classifiers, respectively. In this case, for the k-NN classifier, the mean, RMS, and range features were omitted from the feature vector as shown in Table 12. Furthermore, for J48 DT and RT DT classifiers, both the mean and range were excluded. Additionally, for the RF DT classifier the range feature was removed. The accuracy, AUC, sensitivity, specificity, precision, and the F1 score for the wrapper FS based on RT DT classifier achieved the highest values compared to others. The values recorded 100% accuracy and 1.0 (100%) for the AUC, sensitivity, specificity, precision, and the F1 score, respectively. Whereas, when using the random search strategy, the wrapper RT DT achieved the highest value compared to other classifiers as well. However, this time the highest values were achieved using seven instead of six features as for the best first strategy. Notably, the range feature was omitted for all classifiers as in Table 12. All the values yielded to 100% also as illustrated in Table 4. 

For the digital mammography dream challenge samples, the total number of samples used was 572 as shown in Table 1. The RF DT classifier achieved the highest scores compared to other classifiers as shown in Table 5. The accuracy, AUC, sensitivity, specificity, precision, and the F1 score achieved 98%, 1.0 (100%), 1.0 (100%), 0.962 (96.2%), 0.96 (96%), and 0.98 (98%), respectively as shown in Table 5. Additionally, when using the MCs and their ensemble, the MCs achieved an accuracy, AUC, sensitivity, specificity, precision, and the F1 score of 96.3%, 1.0 (100%), 1.0 (100%), 0.935 (93.5%), 0.93 (93%) and 0.964 (96.4%), respectively. Whereas the Adaboosting and the bagging of these MCs achieved an accuracy of 97.3% and 96.6%, the AUC was 1.0 (100%) for both ensembles and the sensitivity reached 1.0 (100%) as well. Additionally, the specificity and the precision recorded 0.94 (94%) and 0.93 (93%), respectively for both ensembles as illustrated in Table 5. Finally, the F1 score achieved a value of 0.975 (97.5%) and 0.966 (96.6%) for Adaboosting and bagging MCs, respectively. From these results, it was clear that the Adaboosting MC achieved the highest values compared to the others. 

When selecting the features using the best first strategy as it is clear in Table 6 and Table 12, the wrapper FS was based on RF DT classifier with only seven features, excluding the range achieved an accuracy of 98.6%. Whereas, the AUC, sensitivity, specificity, precision, and F1 score achieved 1.0 (100%), 1.0 (100%), 0.974 (97.4%), 0.973 (97.3%), and 0.987 (98.7%), respectively. It is obvious that when selecting features using the best first strategy, the accuracy increased compared to using all the features while the other values maintain the same. 

Conversely, when using the random search FS strategy, the wrapper RF with all the features achieved the highest values compared to other wrappers as shown in Table 7.

On the other hand, when combining the two datasets together, it is obvious from Table 8, in the case of classifying normal and abnormal lesions using individual classifiers, the RF DT classifier achieved the highest accuracy of 98.7% compared to other individual classifiers which achieved an accuracy of 90%, 93.88%, and 97.1% for k-NN, J48 DT, and RT DT classifiers. Moreover, the AUC, sensitivity, specificity, precision, and F1 score for the RF classifier were 0.99 (99%), 0.988 (98.8%), 0.986 (98.6%), 0.965 (96.5%), and 0.972 (97.2%), respectively, which were greater than the other scores performed by the other classifiers.

Furthermore, when the MCS were constructed using k-NN, J48 DT, RF DT, and RT DT classifiers and their ensembles (Adaboosting and bagging), the results showed that these ensemble models have outperformed the performance of these individual classifiers. This proves the concept that usually MCs improves the performance of individual classifiers. In Table 8, the Adaboosting of J48 DT classifier achieved an accuracy of 99.7%, which was greater than the 90%, 99.2%, and 97.8% for the Adaboosting of k-NN, RF DT, and RT DT classifiers, respectively. It was also greater than that of the individual J48 DT classifier (93.88%). The accuracy of Adaboosting J48 DT has also outperformed that of the bagged k-NN (88%), bagged J48 DT (97.4%) and bagged RT DT (98.7%). Additionally, the Adaboosting of J48 DT classifier achieved the highest AUC 1.0 (100%), sensitivity 0.992 (99.2%), specificity 1.0 (100%), precision 0.985 (98.5%), and the F1 score 0.984 (98.4%) compared to the others MCS. The bagging and Adaboosting k-NN achieved AUC (0.934, 0.964), sensitivity (0.85, 0.875), specificity (0.89, 0.9), precision (0.899, 0.978), and the F1 score (0.875, 0.931). Whereas, the bagging and Adaboosting RF DT achieved AUC 0.99 for both cases, sensitivity (0.98, 0.99), specificity (0.98, 0.99), precision (0.986, 0.981), and the F1 score (0.98, 0.892). Finally, the bagging and Adaboosting RT DT achieved AUC (0.99, 0.98), sensitivity (0.98, 0.99), specificity (0.98, 0.96), precision (0.97, 0.98), and the F1 score (0.93, 0.97). 

Additionally, it was clear from Table 8 that the accuracy of the MCS model constructed with the Adaboosting ensembles of (k-NN, J48 DT, RT DT, RF DT) four classifiers reached 99.5%, which was greater than the 90%, 93.88%, 98.7%, and 97.1% of the k-NN, J48 DT, RF DT, and RT DT individual classifiers. This MCS model has also outperformed the performance of MCS constructed using the bagged ensembles of these four classifiers and the MCS constructed using k-NN, J48 DT, RF DT, and RT DT classifiers. Their accuracy reached 98.1% and 97.8% and AUC of 0.99 (99%). Additionally, all the values of the sensitivity, specificity, precision, and the F1 score achieved 0.98 (98%) for both cases. From these results, it is clear that the AUC, sensitivity, specificity, precision, and the F1 score for the MCS constructed using the Adaboosting ensembles of the four classifiers achieved the best results. 

The two FS methods based on the best first and random search were introduced in this paper to select the significant features. Table 9 shows the classification results after the wrapper FS based on the best first searching strategy. The number of selected features was 2, 7, 7 and 7 for k-NN, J48 DT, RF DT, and RT DT classifiers. The range feature was omitted for J48 DT, RF DT, and RT DT classifiers as illustrated in Table 12. However, for the k-NN classifier, only the mean and RMS features were selected. These selected features increased the classification accuracy for the k-NN classifier from 90% to 100%, J48 DT classifier from 93.88% to 98.85%, RF DT classifier from 98.7% to 99.3%, and RT DT classifier from 97.1% to 99.4%. Moreover, these selected features improved the AUC for k-NN classifier from 0.964 (96.4%) to 1.0 (100%), J48 DT classifier from 0.985 (98.5%) to 1.0 (100%), RF DT classifier from 0.990 (99%) to 1.0 (100%), and RT DT classifier from 0.970 (97%) to 0.995 (99.5%). In addition, the reduced number of features increased the sensitivity, specificity, precision, and the F1 score as shown in Table 9. 

On the other hand, when using the second wrapper FS method based on the random search using k-NN, RF DT, and RT DT classifiers, the number of features reduced to 6, 7, and 7 features. For the k-NN classifier, the range and maximum features were omitted by the FS strategy. On the other hand, for the RT DT classifier, the entropy and range were removed by the FS method. The accuracies achieved were 99.7%, 99.3%, and 99.4%, which proved to be greater than 90%, 98.7%, and 97.1% of the full model k-NN, RF DT, and RT DT classifiers as illustrated in Table 10. Moreover, the AUC, sensitivity, specificity, precision, and the F1 score for k-NN, RF DT, and RT DT classifiers after FS were better than that of the full model as in Table 10. 

**Figure 7:**
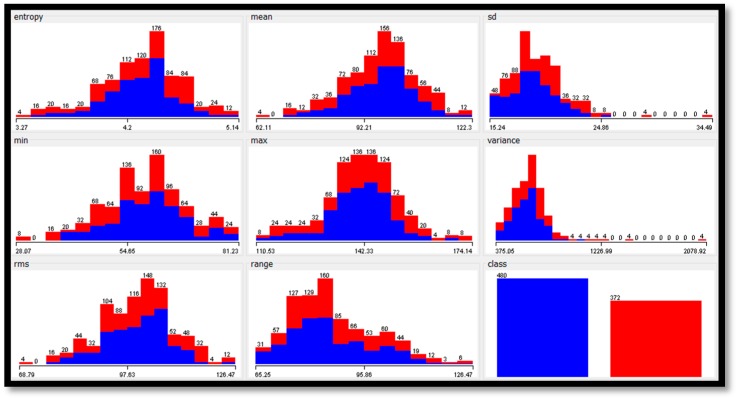
The histograms of features values of the MIAS dataset.

**Figure 8:**
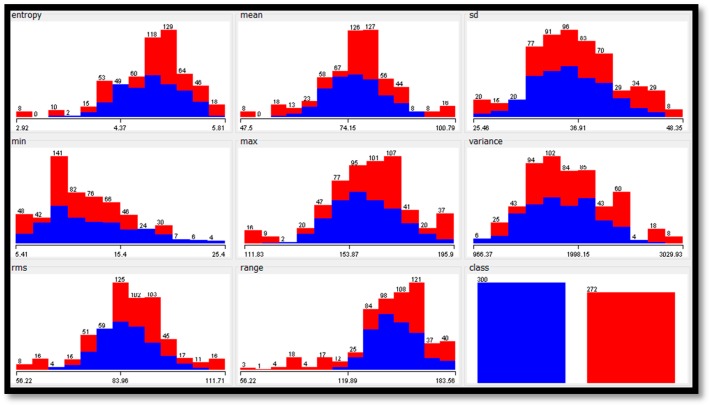
The histograms of features values of the digital mammography dream challenge dataset.

**Figure 9:**
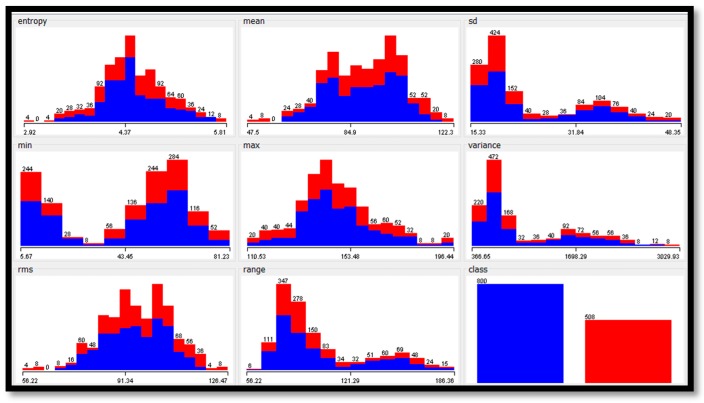
The histograms of features values of the combination of the MIAS and digital mammography dream challenge datasets.

To analyze the features extracted from the datasets, Table 13 shows the statistical analyses for each feature in each dataset. Furthermore, Figure 7, Figure 8 and Figure 9 show the histogram representation for each feature of the breast cancer datasets. 

Finally, the proposed CAD system has been compared with other papers in the field that have the same conditions to prove the efficiency of the proposed method as shown in Table 11. Regarding the MIAS dataset, it was clear that the results have shown that the proposed CAD system recorded the highest accuracy and AUC compared to El-Toukhy et al. [47], Beura et al. [15], Pawar and Talbar [16], Phadke and Rege [48], and Mohanty et. al. [17]. Moreover, when combining the two datasets together, the accuracies achieved using the Adaboosting of J48 DT, wrapper k-NN based on a random search and wrapper k-NN based on the best first search were 99.7%, 99.7%, and 100%. These accuracies were greater than 95.84% and 95.98% of the method proposed by El-Toukhy et al. [47] using the wavelet and curvelet feature extraction methods.

The accuracies of the proposed method were better than that in Beura et al. [15], which had an accuracy of 98% and 98.8% with MIAS and the digital database for screening mammography (DDSM) [8], respectively. The accuracies were greater than the methods in Pawar and Talbar [16] and Mohanty et al. [17] which applied FS approaches to reach an accuracy of 89.47% and 97.86%. Moreover, the proposed CAD system has AUC of 1.0, 0.995, and 1.0 using the Adaboosting of J48 DT, wrapper k-NN based on random search and wrapper k-NN based on the best first search. This was greater than the 0.98 and 0.978 of the method proposed in Fu et al. [14] who used the sequential feature selection algorithm (SFS). 

Nowadays, deep learning is considered the fastest growing field in machine learning techniques [49,50,51,52]. Therefore, the future work will be applying several deep learning techniques to classify the breast cancer lesions. 

## 6. Conclusions

The goal of this work was to classify the normal and abnormal breast tissues in mammograms. A new CAD system was proposed. The breast was suppressed from any artifacts and the pectoral muscle was eliminated as they appear, approximately, with a similar density as the dense tissues in the mammogram image. In the feature extraction step, some statistical features were extracted and used to construct several individuals, ensemble, and multiple classifier systems. The suggested CAD system was capable of classifying normal and abnormal breast tissues in mammograms. It considered the influence of combing two datasets on classification accuracy. It also studied the effect of using multiple classifier systems with different structures combined with the feature selection on classification accuracy. The proposed CAD system achieved a classification accuracy of 99.5% (using an individual RT DT classifier) and 97.55% (using an individual RF DT classifier) for MIAS and Dream Challenge datasets, respectively. The classification accuracy reached 98.7% using an individual RF DT classifier for MIAS and Dream Challenge datasets when combined together. This is better than the 97.55% of the Dream Challenge dataset. Moreover, the CAD system, constructed using the ensemble classifiers of the same type and multiple classifiers of different types, outperformed the classification results of the individual classifiers for both MIAS and Dream Challenge datasets when used separately and combined together. On the other hand, the features selected using the best first and the random search methods of the CAD system have improved the classification performance of the classification models for both MIAS and Dream Challenge datasets when used separately and combined together. Finally, the proposed CAD system results outperformed the performance of several previous CAD systems that have appeared in the literature. The proposed CAD system was able to classify the lesions completely, as the accuracy using the best first wrapper FS based on k-NN was 100% in the case of combining the two datasets. Therefore, the proposed CAD system could be considered as a powerful tool to detect and classify abnormalities in the breast. 

## Figures and Tables

**Table 1:** The number of samples used for each dataset.

	Normal	Abnormal	Total
MIAS	480	372	852
The digital mammography dream challenge	300	272	572
The combination of the two datasets	800	508	1308

**Table 2:** Classification results of the four individual classifiers and the multiple classifier systems (MCS) constructed with all the four classifiers and their ensembles for the MIAS dataset.

Classifier	Accuracy	AUC	Sensitivity	Specificity	Precision	F1 Score
k-NN	88.9%	0.951	0.884	0.895	0.896	0.89
Adaboosting k-NN	88.9%	0.951	0.884	0.895	0.896	0.89
Bagged k-NN	86.7%	0.935	0.836	0.894	0.903	0.869
J48 DT	98.2%	0.994	0.981	0.99	0.99	0.986
Adaboosting J48 DT	100%	1.000	1.000	1.000	1.000	1.000
Bagged J48 DT	97.7%	0.996	0.952	0.99	0.99	0.971
RF DT	100%	1.000	1.000	1.000	1.000	1.000
Adaboosting RF DT	99.6%	1.000	0.991	1.000	1.000	0.996
Bagged RF DT	98.4%	0.999	0.971	0.99	0.99	0.981
RT DT	99.5%	0.995	0.99	0.99	0.99	0.99
Adaboosting RT DT	99.4%	0.994	0.99	0.99	0.99	0.99
Bagged RT DT	98.7%	1.000	0.971	0.99	0.99	0.981
k-NN + J48 DT + RF DT + RT DT (averaging probabilities)	99.4%	1.000	0.981	0.99	0.99	0.986
Adaboosting (k-NN + J48 DT + RF DT + RT DT (averaging probabilities))	99.8%	1.000	1.000	0.991	0.99	0.995
Bagging (k-NN + J48 DT + RF DT + RT DT (averaging probabilities))	98.7%	0.998	0.961	0.98	0.98	0.971

**Table 3:** The classification results before and after the feature selection results using best first (stepwise forward and backward search strategy) for the MIAS dataset.

Classifier	Number of Features	Accuracy	AUC	Sensitivity	Specificity	Precision	F1 Score
k-NN (without FS)	8	88.9%	0.951	0.884	0.895	0.896	0.89
Wrapper k-NN after (with FS)	5	99.5%	1.000	1.000	0.992	0.992	0.996
J48 DT (without FS)	8	98.2%	0.994	0.981	0.99	0.99	0.986
Wrapper J48 DT (with FS)	6	98.5%	0.994	0.98	0.993	0.98	0.98
RF DT (without FS)	8	100%	1.000	1.000	1.000	1.000	1.000
Wrapper RF DT (with FS)	7	99.6%	1.000	0.991	1.000	1.000	0.996
RT DT (without FS)	8	99.5%	0.995	0.99	0.99	0.99	0.99
Wrapper RT DT (with FS)	6	100%	1.000	1.000	1.000	1.000	1.000

**Table 4:** The classification results before and after the feature selection results using the random search method for the MIAS dataset.

Classifier	Number of Features	Accuracy	AUC	Sensitivity	Specificity	Precision	F1 Score
k-NN (without FS)	8	88.9%	0.951	0.884	0.895	0.896	0.89
Wrapper k-NN (with FS)	7	99.5%	1.000	1.000	0.991	0.99	0.995
J48 DT (without FS)	8	98.2%	0.994	0.981	0.99	0.99	0.986
Wrapper J48 DT (with FS)	8	98.8%	0.994	0.981	0.99	0.99	0.986
RF DT (without FS)	8	100%	1.000	1.000	1.000	1.000	1.000
Wrapper RF DT (with FS)	7	99.6%	1.000	0.991	1.000	1.000	0.996
RT DT (without FS)	8	99.5%	0.995	0.99	0.99	0.99	0.99
Wrapper RT DT (with FS)	7	100%	1.000	1.000	1.000	1.000	1.000

**Table 5:** The classification results of the four individual classifiers and the MCS constructed with all the four classifiers and their ensembles digital mammography dream challenge dataset.

Classifier	Accuracy	AUC	Sensitivity	Specificity	Precision	F1 Score
k-NN	95.9%	0.99	1.000	0.929	0.924	0.961
Adaboosting k-NN	95%	0.99	1.000	0.929	0.924	0.961
Bagged k-NN	94.4%	0.98	1.000	0.855	0.83	0.908
J48 DT	88%	0.922	0.991	0.817	0.777	0.872
Adaboosting J48 DT	96.3%	1.000	1.000	0.935	0.93	0.964
Bagged J48 DT	94.5%	0.996	1.000	0.906	0.896	0.946
RF DT	98%	1.000	1.000	0.962	0.96	0.98
Adaboosting RF DT	97.2%	1.000	1.000	0.958	0.956	0.978
Bagged RF DT	95.8%	1.000	1.000	0.926	0.92	0.959
RT DT	93.8%	0.942	1.000	0.893	0.88	0.937
Adaboosting RT DT	94.5%	0.948	0.996	0.909	0.9	0.946
Bagged RT DT	96.5%	0.999	1.000	0.938	0.933	0.966
k-NN + J48 DT + RF DT + RT DT (averaging probabilities)	96.3%	1.000	1.000	0.935	0.93	0.964
Adaboosting (k-NN + J48 DT + RF DT + RT DT (averaging probabilities))	97.3%	1.000	1.000	0.944	0.94	0.97
Bagging (k-NN + J48 DT + RF DT + RT DT (averaging probabilities))	96.6%	1.000	1.000	0.938	0.933	0.966

**Table 6:** The classification results before and after feature selection results using best first (stepwise forward and backward search strategy) for the digital mammography dream challenge.

Classifier	Number of features	Accuracy	AUC	Sensitivity	Specificity	Precision	F1 Score
k-NN (without FS)	8	96%	0.99	1.000	0.929	0.924	0.961
Wrapper k-NN (with FS)	7	96.3%	0.991	1.000	0.935	0.93	0.964
J48 DT (without FS)	8	87.9%	0.922	1.000	0.929	0.924	0.961
Wrapper J48 DT (with FS)	7	90.73%	0.949	0.83	0.993	0.83	0.83
RF DT (without FS)	8	98%	1.000	1.000	0.962	0.96	0.98
Wrapper RF DT (with FS)	7	98.6%	1.000	1.000	0.974	0.973	0.987
RT DT (without FS)	8	93.88%	0.942	1.000	0.893	0.88	0.937
Wrapper RT DT (with FS)	3	96.1%	0.963	1.000	0.926	0.92	0.959

**Table 7:** The classification results before and after feature selection results using the random search method for the digital mammography dream challenge.

Classifier	Number of Selected Features	Accuracy	AUC	Sensitivity	Specificity	Precision	F1 Score
k-NN (without FS)	8	96%	0.99	1.000	0.929	0.924	0.961
Wrapper k-NN (with FS)	5	96.85%	1.000	1.000	0.944	0.94	0.97
J48 DT (without FS)	8	88%	0.922	1.000	0.929	0.924	0.961
Wrapper J48 DT (with FS)	4	95.1%	0.96	1.000	0.915	0.907	0.952
RF DT (without FS)	8	98%	1.000	1.000	0.962	0.96	0.98
Wrapper RF DT (with FS)	8	98%	1.000	1.000	0.962	0.96	0.98
RT DT (without FS)	8	93.88%	0.942	1.000	0.893	0.88	0.937
Wrapper RT DT (with FS)	6	95%	0.951	0.996	0.915	0.907	0.95

**Table 8:** The classification results of the four individual classifiers and the MCS constructed with all the four classifiers and their ensembles for the combination of the two datasets.

Classifier	Accuracy	AUC	Sensitivity	Specificity	Precision	F1 Score
k-NN	90%	0.964	0.875	0.907	0.91	0.893
Adaboosting k-NN	88%	0.934	0.854	0.894	0.899	0.876
Bagged k-NN	90%	0.964	0.875	0.907	0.91	0.893
J48 DT	93.88%	0.985	0.89	0.976	0.978	0.932
Adaboosting J48 DT	97.4%	0.996	0.966	0.98	0.98	0.973
Bagged J48 DT	99.7%	1.000	0.993	1.000	1.000	0.997
RF DT	98.7%	0.99	0.988	0.987	0.986	0.987
Adaboosting RF DT	98.5%	0.99	0.985	0.985	0.985	0.985
Bagged RF DT	99.2%	0.99	0.993	0.994	0.994	0.994
RT DT	97.1%	0.97	0.98	0.966	0.965	0.973
Adaboosting RT DT	98.7%	0.99	0.988	0.987	0.986	0.987
Bagged RT DT	97.8%	0.98	0.992	0.97	0.969	0.981
k-NN + J48 DT + RF DT + RT DT (averaging probabilities)	97.8%	0.99	0.98	0.979	0.978	0.979
Adaboosting (k-NN + J48 DT + RF DT + RT DT (averaging probabilities))	99.5%	1.000	1.000	0.994	0.993	0.997
Bagging (k-NN + J48 DT + RF DT + RT DT (averaging probabilities))	98.1%	0.99	0.982	0.982	0.981	0.982

**Table 9:** The classification results before and after feature selection results using best first (stepwise forward and backward search strategy) for the combination of the two datasets.

Classifier	Number of Features	Accuracy	AUC	Sensitivity	Specificity	Precision	F1 Score
k-NN (without FS)	8	90%	0.964	0.875	0.907	0.91	0.893
Wrapper k-NN (with FS)	2	100%	1.000	1.000	1.000	1.000	1.000
J48 DT (without FS)	8	93.88%	0.985	0.89	0.976	0.978	0.932
Wrapper J48 DT (with FS)	7	98.85%	1.000	0.994	0.986	0.985	0.99
RF DT (without FS)	8	98.7%	0.99	0.988	0.987	0.986	0.987
Wrapper RF DT (with FS)	7	99.3%	1.000	0.989	0.996	0.996	0.993
RT DT (without FS)	8	97.1%	0.97	0.98	0.966	0.965	0.973
Wrapper RT DT (with FS)	7	99.4%	0.995	1.000	0.991	0.99	0.995

**Table 10:** The classification results before and after feature selection results using the random search method for the combination of the two datasets.

Classifier	Number of Features	Accuracy	AUC	Sensitivity	Specificity	Precision	F1 Score
k-NN (without FS)	8	90%	0.964	0.875	0.907	0.91	0.893
Wrapper k-NN (with FS)	6	99.7%	0.995	1.000	0.996	0.995	0.998
J48 DT (without FS)	8	93.88%	0.985	0.89	0.976	0.978	0.932
Wrapper J48 DT (with FS)	8	93.88%	0.985	0.883	0.976	0.978	0.929
RF DT (without FS)	8	98.7%	0.99	0.988	0.987	0.986	0.987
Wrapper RF DT (with FS)	7	99.3%	1.000	0.989	0.996	0.996	0.993
RT DT (without FS)	8	97.1%	0.97	0.98	0.966	0.965	0.973
Wrapper RT DT (with FS)	6	99.4%	0.99	0.996	1.000	1.000	0.998

**Table 11:** Classification results for different breast classification methods using different classifiers.

Reference	Contribution	Data Set	Accuracy	AUC
Fu et al. [14]	Features were extracted from both the spatial domain and spectral domain, then selected by sequential forward search algorithm, and classified by SVM and GRNN	Nijmegen University Hospital (Netherlands) database	-	0.98 0.978
El Toukhy et al. [47]	Wavelet for feature extraction and SVM to classify normal and abnormal Curvelet transform for feature extraction and SVM to classify normal and abnormal	MIAS	95.84% 95.98%	-
Beura et al. [15]	Grey level co-occurrence matrix (GLCM) and DWT to extract the texture features, features were selected using the filter methods namely two-sample t-test and F-test, classified by BPN	MIAS DDSM	98% 98.8%	-
Pawar and Talbar [16]	Features were extracted using the wavelet co-occurrence, selected using the wrapper method, and classified by the fuzzy classifier	MIAS	89.47%	-
Phadke and Rege [48]	Local and global feature extraction and SVM to classify normal and abnormal	MIAS	93.1%	-
Mohanty et al. [17]	Contourlet transform feature extraction, features were selected using wrapper forest optimization algorithm, and Naïve Bayes classifier for classifying normal and abnormal lesions	MIAS	97.86%	-
The proposed System	Statistical feature extraction and Adaboosting J48 DT	MIAS	100%	1.000
	Statistical feature extraction and RF DT		100%	1.000
	Statistical feature extraction, best first FS, and wrapper RT DT		100%	1.000
	Statistical feature extraction, random search FS, and wrapper RT DT		100%	1.000
	Statistical feature extraction and RF DT	Dream Challenge	98%	1.000
	Statistical feature extraction, best first FS, wrapper RF DT		98.07%	1.000
	Statistical feature extraction, random search FS, wrapper RF DT		98%	1.000
	Statistical feature extraction and Adaboosting J48 DT	MIAS and Dream Challenge	99.7%	1.000
	Statistical feature extraction, random search FS, wrapper k-NN		99.7%	0.995
	Statistical feature extraction, best first FS, wrapper k-NN		100%	1.000

**Table 12:** The name of the unselected features for each FS method for breast cancer datasets.

Classifier	Number of Selected Features	Name of Unselected Features
**MIAS Dataset—Best First FS Strategy**		
Wrapper k-NN	5	Mean, RMS, and range
Wrapper J48 DT	6	Mean and range
Wrapper RF DT	7	Range
Wrapper RT DT	6	Mean and range
**MIAS Dataset—Random Search FS Strategy**		
Wrapper k-NN	7	Range
Wrapper J48 DT	7	Range
Wrapper RF DT	7	Range
Wrapper RT DT	7	Range
**Digital Mammography Dream Challenge Dataset—Best First FS Strategy**		
Wrapper k-NN	7	Range
Wrapper J48 DT	7	Range
Wrapper RF DT	7	Range
Wrapper RT DT	3	Entropy, variance, standard deviation, RMS, range
**Digital Mammography Dream Challenge Dataset—Random Search FS Strategy**		
Wrapper k-NN	5	Entropy, variance, and range
Wrapper J48 DT	4	Entropy, mean, max, and range
Wrapper RF DT	8	-
Wrapper RT DT	6	Mean and standard deviation
**MIAS + Dream Challenge Dataset—Best First FS Strategy**		
Wrapper k-NN	2	Entropy, variance, standard deviation, minimum, maximum, and range
Wrapper J48 DT	7	Range
Wrapper RF DT	7	Range
Wrapper RT DT	7	Range
**MIAS + Digital Mammography Dream Challenge Dataset—Random Search FS Strategy**		
Wrapper k-NN	6	Maximum and range
Wrapper J48 DT	8	-
Wrapper RF DT	7	Range
Wrapper RT DT	6	Entropy and Range

**Table 13:** Statistical analysis for each feature of the breast cancer datasets.

Feature	Statistical Analysis			
	Minimum	Maximum	Mean	Standard Deviation
**MIAS Dataset**				
Entropy	3.27	5.13	4.32	0.33
Mean	62.11	122.29	96.73	10.08
Standard Deviation	15.23	34.48	19.34	2.41
Minimum	28.07	81.23	58.84	9.84
Maximum	110.52	174.14	141.43	10.89
Variance	375.05	2078.91	656.39	176.06
RMS	68.78	126.47	100.36	9.70
Range	65.25	126.47	87.03	11.76
**Digital Mammography Dream Challenge Dataset**				
Entropy	2.92	5.81	4.79	0.51
Mean	47.5	100.79	76.57	9.17
Standard Deviation	25.46	48.35	36.64	4.69
Minimum	5.4	25.4	12.2	4.02
Maximum	111.82	195.9	161.71	16.814
Variance	966.37	3029.92	1899.42	394.69
RMS	56.21	111.7	86.46	9.87
Range	56.21	183.56	145.68	21.67
**MIAS + Digital Mammography Dream Challenge Dataset**				
Entropy	2.92	5.81	4.47	0.45
Mean	47.5	122.29	90.17	13.19
Standard Deviation	15.33	48.35	24.72	8.49
Minimum	5.66	81.23	44.01	22.63
Maximum	110.52	196.44	147.66	16
Variance	366.64	3029.92	1035.48	613.02
RMS	56.21	126.47	95.73	11.41
Range	56.21	186.36	101.66	29.2
